# Autoimmune Hemolytic Anemia preceding the Diagnosis of Primary Central Nervous System Lymphoma 

**Published:** 2015-03-15

**Authors:** H Farhangi, N Sharifi, H Ahanchian, A Izanloo

**Affiliations:** 1 Department of Pediatric Hematology-Oncology, Dr Sheikh Pediatric Hospital, Mashhad University of Medical Sciences, Mashhad, Iran.; 2 Department of Pathology, Faculty of Medicine, Mashhad University of Medical Sciences, Mashhad, Iran.; 3 Department of Pediatric Immunology, Ghaem Hospital Mashhad University of Medical Sciences, Mashhad, Iran.; 4 M.S.C in Medical Education, Faculty of Paramedical Sciences, Mashhad University of Medical Sciences, Mashhad, Iran.

**Keywords:** Anemia, Lymphoma, Nervous System, MRI

## Abstract

In this study, a 2.5-year-old boy suffering from a febrile seizure with normal laboratory tests and a history of immune hemolytic anemia was examined. Brain MRI demonstrated some tumors in the frontal, parietal, and occipital lobe that corroborated the pathology results of primary central nervous system lymphoma for the patient. The patient was treated with high-dose of Methotrexate. Our result suggested regular and careful monitoring of patients with autoimmune hemolytic anemia in order to control the manifestations of concomitant disease such as lymphoma that may develop later.

## Introduction

Primary central nervous system lymphoma (PCNSL) is a rare disease in children, constituting about 1 to 3 percent of all central nervous system malignancies (1).In fact, it is a non-hodgkin lymphoma which arises from parenchyma of the brain, eyes, meninges or spinal cord in the absence of a systemic disease (2). In patients with immunodeficiency, the average age of PCNSL diagnosis is 53 to 57 years with a male to female ratio of 1.2- 1.7 (3). In retrospective follow-up examinations, brain masses in supratentorial (87%) and front parietal lobes (39%) (4) were shown in patients with immunodeficiency and PCNSL.

For decades, radiotherapy was the main treatment for the PCNSL patients, but due to its significantly adverse effects on children's brain development and Its poor treatment outcomes, it is no longer adopted. 

Current studies on a series of patients omit that various chemotherapeutic methods, particularly the use of high-dose methotrexate, are very effective in the treatment of the disease and can improved its prognosis. 

Prospective studies for the diagnosis and the treatment of PCNSL were not possible because of its rarity. In the performed studies, the methods of PCNSL treatment and prognosis were mainly based on the case reports and adult studies (6, 7). Therefore, the first case of autoimmune hemolytic anemia before the diagnosis of the disease in a 2.5-year-old boy was investigated as a caser report. 

## Case report

**Figure 1:**
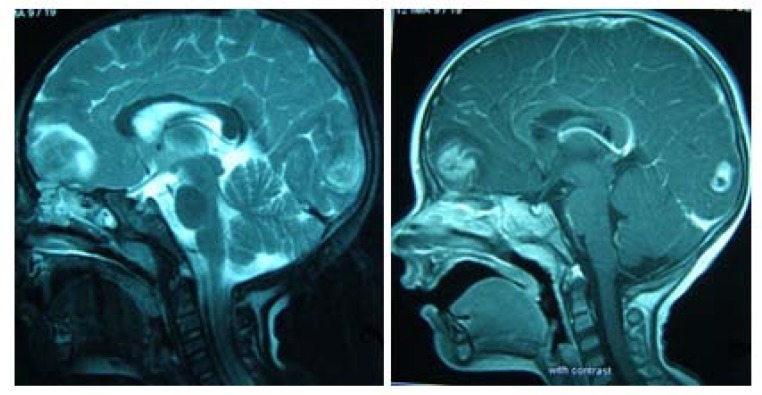
Sagittal T_1_ weighted MRI showing the primary lymphoma of central nervous system involving the occipital and frontal regions, with post contrast images demonstrating an intense enhancement

**Figure 2:**
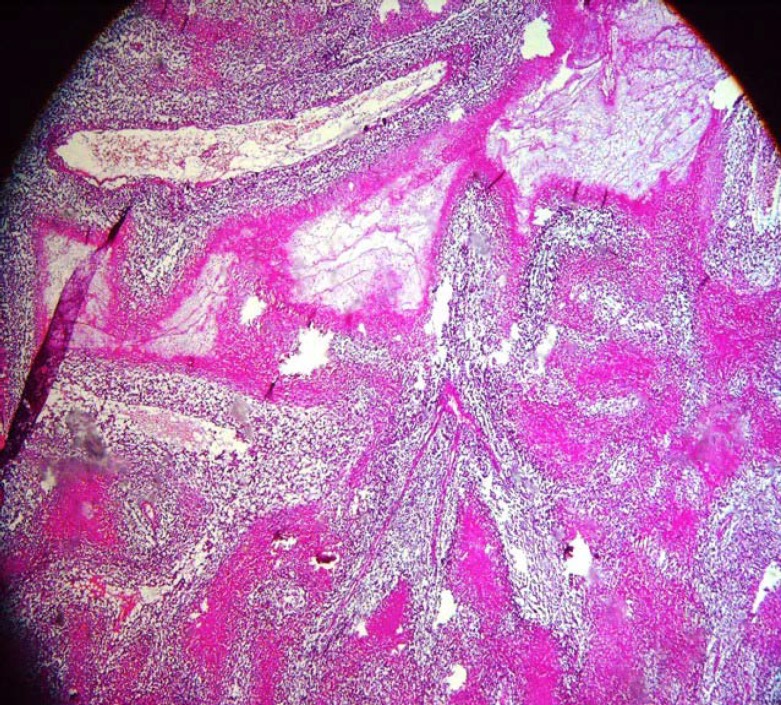
Microscopic view of tumor lesion which consists of malignant small-round cell tumors with extensive necrosis

**Figure 3:**
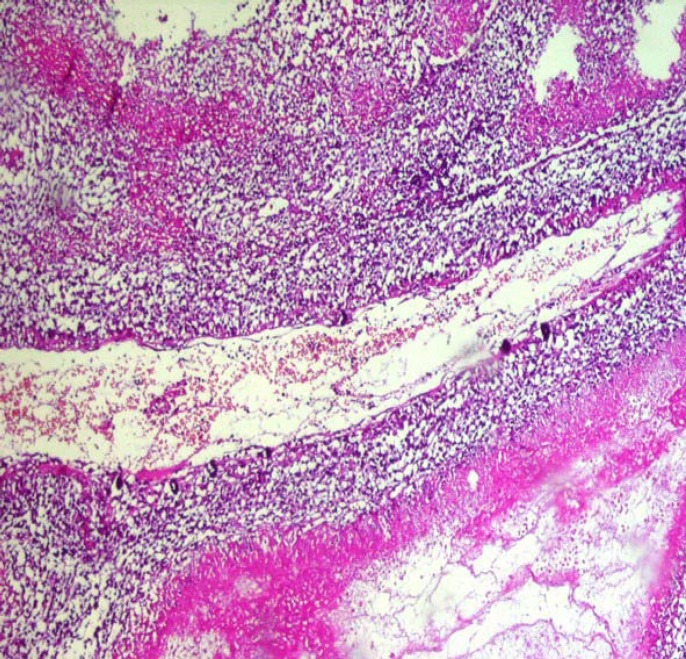
Microscopic view shows perivascular condensation of tumoral cells

**Figure 4:**
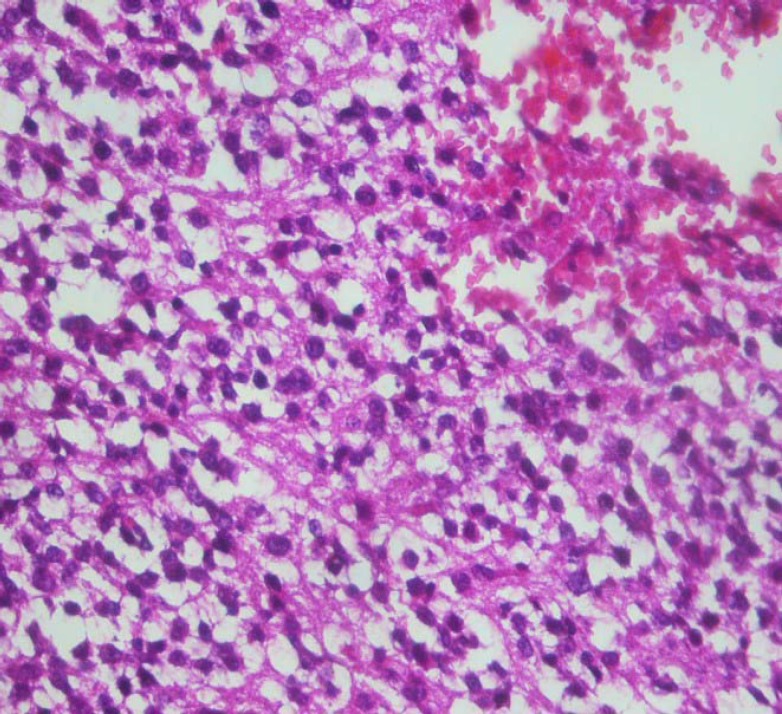
Microscopic view of a tumoral lesion with small and medium size hyperchromaticnucleialong with someacidophil cytoplasm and foci of necrosis

A 2.5- year old boy with a febrile seizure was admitted to the hospital. His healthy parents were first degree relatives and the patient had normal growth and developmental parameters. Physical examination revealed a low level of consciousness, left abducens nerve palsy, headache, incoordination and speech disorder. He was previously admitted to the hospital twice due to Immune Hemolytic anemiaonce at the age of 2 months, and later when he was 12 months old. He was treated with prednisolone. Laboratory results for complete blood count, sodium, potassium, blood sugar, calcium, urea, creatinine, liver function and albumin were normal. Direct coombs test was positive and the serum Immune globulin levels were normal, whenever there were anactivehemolysis.ELISA test results for Epstein–Barr virus and acquired immunodeficiency virus (HIV) were negative. Given his persistent drowsiness and recurrent seizures, brain CT scan was performed and a mass lesion in the parietal lobe was discovered. Further MRI investigation revealed multi-centric mass lesions in frontal, parietal and occipital lobes (Figure 1). Being suspected of PCNSL or cerebral metastases, the patient underwent open brain biopsy. Histopathologic examination of the brain lesion demonstrated an irregular fragmented tumorallesion with extensive vascular along with parenchymal necrosis with pleomorphic and hyperchrom nuclei. Instances of clear perinuclear hollow, focal calcification, and stromal fibrillar appearance were also observed (Figures2, 3&4). In the immunohistochemical study, the neoplastic cells were labeled Leukocyte Common Antigen (LCA) and CD20.Negative reactivity was also labeled glial fibrillary acid protein. These findings were consistent with the studies on PCNSL– diffuse large B cell lymphoma. There was nosign of systemic disease. Bone marrow aspiration, biopsy, CSF cytospin, abdomino-pelvic ultrasonographyand skeletal bone survey was all normal. Moreover, all immune system evaluations such as Immunoglobulins, B cell count and function, T cell count, subtypes and function, serum complement and neutrophils count and function were normal. 

The patient was treated with courses of high dose METHOTREXATE (8 gr / m2) according to NABTT 96-07 protocol. He did not receive intrathecal injection and radiotherapy to the CNS. He was in good health for 42 months after the termination of the treatment. Currently, the patient has right hemiparesis, but he is able to walk. His speech is slow and intense (Cerebellar dysarthria).

## Discussion

The results of a 19-year-long study in Japan show that out of 596 cases of PCNSL, only 9 cases (1.5%) happen in children (6). In the study of BFM group (Berlin-Frankfort- Munster) on 2311 children with NHL, only 10 patients (0.43%) were diagnosed with PCNSL (8). Patients with congenital or acquired immune deficiency are more at the risk of this disease; although, in most children with PCNSL, immunodeficiency has not been reported. 

The results of this study indicate that immunodeficiency – whether congenital or acquired - may increase the risk of diseases such as PCNSL. In case series studies on children, the predominance of the disease in males and a median age of 12 years and a minimum age of 2 years were reported (6,8). In this study, a boy with a minimum age of two and a half is studied, which is in line with the above results.

As we know, MRI scanning is very useful for the diagnosis of PCNSL and the lesion is described as hypointense in T1 images and hypointense to isointense in T2 images. After gadolinium injection, the accumulation is dispersed in the lesion (9). In patients with immunodeficiency, a tendency toward multifocal infiltration and basal ganglia involvement is more evident in MRI scanning. Also, after the injection of contrast material in patients with immunodeficiency, the contrast material accumulation appeared heterogeneously and the ring enhancement became more apparent (10). 

Stereotactic biopsy is the standard golden method for diagnosing PCNSL. The present case study is in line with Abla et al. research which considered diffuse large B cell lymphoma (DLBCL) as the most common type and anaplastic large cell lymphoma (ALCL), Lymphoblastic lymphoma and burkitt-like lymphoma as types with lower incidence(6).

Finally, PCNSL is an unusual form of NHL that is restricted to the CNS. Although, accompanied with focal neurologic symptoms, it is characterized pathologically by diffuse infiltration of the brain. Since clinical presentations and radiographic features in both immunodeficient and non-immunodeficient patients are similar and nonspecific, it is not possible to ascertain whether the patient has developed the disease out of an immunodificient background or not. A brain MRI revealing a homogenously enhancing single lesion is highly suggestive of PCNSL in immunocompetent patients, whereas multiple ring-enhancing lesions are more common in immunodeficient patients. A brain biopsy remains the golden standard for PCNSL diagnosis in all patients. PCNSL is sensitive to corticostroids, radiotherapy and chemotherapy. High-dose methotrexate regimens are the cornerstone of multimodality therapy. 

## Conclusions

The findings of this study suggest a regular and careful monitoring of patients with autoimmune hemolytic anemia for incidence of concomitant disease such as lymphoma that may develop later.
